# Evaluation and Remediation of Data Quality for Drilling-Fluid Property Online Monitoring

**DOI:** 10.3390/s26217009

**Published:** 2026-11-05

**Authors:** Qi Lu, Zhilin Li, Dongxiao Pang

**Affiliations:** Drilling and Production Engineering Technology Research Institute, Chuanqing Drilling Engineering Co., Ltd., CNPC, Chengdu 610000, China; lizl_ccde@cnpc.com.cn (Z.L.); dongxx_zyy@cnpc.com.cn (D.P.)

**Keywords:** deep well, drilling fluid, online monitoring, drilling-fluid sensor, data quality evaluation, missing-value treatment, outlier identification, signal enhancement

## Abstract

**Highlights:**

**What are the main findings?**

- Six metrics quantify the quality of drilling-fluid sensor data.
- Tiered remediation invalidates long gap windows and suppresses their triggering.

**What are the implications of the main findings?**

- An operation mask separates sensor artefacts from genuine transients.
- Warning models trained on the remediated data gave a mean lead time of 1.8–2.8 h in the evaluated field case.

**Abstract:**

Missing values, outliers, out-of-order timestamps, unit inconsistencies and baseline drift degrade online monitoring data on drilling-fluid properties in deep wells. We validate an evaluation–marking–remediation–validation workflow on a multi-source database of about 500,000 business records and on 150,000 instrument and replay records that are registered separately by source and are not summed, from five wells. Six metrics describe data quality: missing rate, out-of-order rate, outlier rate, unit consistency, baseline drift and noise. Missing values are treated in tiers: short gaps are interpolated, medium gaps are interpolated as last-observation-carried-forward with a mask, and long gaps invalidate the window. Offline filling uses forward filling, linear interpolation or random forest prediction. MAD/Hampel detection with operating-condition matching separates sensor artefacts, operational transients and genuine anomalies. Signal enhancement combines robust denoising, multi-scale fusion and a trend-confidence threshold. On the same replay dataset, the missing rate fell from 6.8% to 0.7%, the outlier rate decreased from 3.6% to 0.8%, the baseline-drift index fell from 1.47 to 0.61, the noise level decreased by 40.8%, and the usable-record ratio rose from 77.0% to 98.0%. On a fixed judgement set of 55 genuine events and 164 negative exposure intervals, the operator that retains operations and multi-metric co-movement recovered 55 of 55 genuine events at a negative-interval false alarm rate of 25.61%, against 0% recall for the 3-sigma rule and 54.55% recall for Hampel alone; this comparison covers simplified cleaning operators and is not equivalent to the complete remediation chain. Warning models trained on the remediated data reached an F1 of 93.0–94.0% and lead times of 1.8–2.8 h on the replay test dataset. The raw data replay and the field application are two different data sources and are not compared as an effect of remediation.

## 1. Introduction

Accurate real-time knowledge of drilling-fluid performance directly affects wellbore stability, the rate of penetration and operational safety in deep wells [1,2,3]. Several measurement platforms are now in routine use: automated rheology and density devices [4], continuous online monitoring systems [5,6], modular deep-water units [7,8], remote operation centres linked to hydraulic models [9,10,11,12], and automated drilling-fluid management systems [13,14,15,16,17]. The real-time measurement and data-driven modelling of drilling-fluid rheology form a distinct line of work in sensing and measurement [18,19], in which anomaly detection and the quality remediation of sensor data are key steps [20,21]. As monitoring scale and sampling frequency grow, the accumulated field data can support performance analysis and intelligent early warning.

Field monitoring data are not yet clean enough to support analysis and early warning directly. Online detection equipment works for long periods under vibration, electrical surges, electromagnetic interference, fluctuating air supply, changing sampling-pump conditions and unstable communication. Missing values, outliers, out-of-order timestamps, unit inconsistencies, baseline drift and elevated noise are therefore common [10,11,22,23]. These defects propagate into downstream analysis: improper imputation creates spurious trends, untreated outliers raise the false alarm rate, and over-smoothing hides genuine transients. Drilling-fluid monitoring involves weak signals, strong noise and non-stationary conditions, so data quality remediation sets the achievable warning performance [24,25].

Existing studies improve data quality mainly at the sensor and measurement levels [10,11,22,23] or parse and digitise unstructured daily reports [26]. Multivariate statistical process monitoring can detect abnormal states, but it usually assumes complete data and stationary conditions [24]. General methods for missing-value imputation and anomaly detection target generic time series and do not fully use the continuity, the physical feasibility domain and the operational signatures of drilling-fluid parameters. There is therefore a clear engineering need for a data quality evaluation and remediation method that is operational, verifiable and able to distinguish data anomalies from genuine transients.

This paper addresses data remediation for 500,000 multi-source heterogeneous monitoring records from five wells in the Sichuan–Chongqing area. We (1) establish an evaluation index system comprising the missing rate, out-of-order rate, outlier rate, unit consistency, baseline-drift index and noise level; (2) propose tiered missing-value remediation with physical feasibility domain constraints; (3) establish a differentiated outlier-treatment strategy based on MAD/Hampel detection and operating-condition matching; and (4) enhance the signals by robust denoising, multi-scale fusion, a trend-confidence threshold and interaction features. Field tests and downstream warning models then validate the remediation effect.

## 2. Materials and Methods

### 2.1. Data Sources and Data Characteristics

The data are registered separately by source. (1) Multi-source database: About 500,000 business records covering engineering parameters, performance parameters, contamination and warning records; this figure describes the scale of the information resource and is not used as an independent sample size. (2) Instrument and replay data: 150,000 records from five wells in the Sichuan–Chongqing area, used as the quality replay set. (3) Engineering parameter data, sampled once every 30 min. (4) Treatment-record data: event-triggered field records of additive type, dosage, operating steps and treatment effect. Because different sources may contain copies or derived records of the same observation, the database size and the task sample sizes are reported separately and are not summed. Routine performance parameter sampling is once every 15 min to once per hour; the 5 min interval used during the field campaign was a special configuration requested by the operator, in which final gel strength was not measured, and the shortest achievable cycle is 15 min when final gel strength is included. Data remediation is the core of this paper; the three warning models serve only as validation tasks. Data volumes and split ratios are given in Table 1, the composition of data sources and types is given in Table 2. And model performance is given in Table 12.

#### Dataset Partition and Leakage Control

All three warning tasks use field monitoring data from five wells in the Sichuan–Chongqing area, divided 7:2:1 by well group and by time into training, validation and evaluation subsets: training was conducted from 1 January 2024 to 31 December 2024; validation was conducted from 4 January 2025 to 28 February 2025; and evaluation was conducted from 4 March 2025 to the end of each task period (CO_2_ to 31 October 2025, salt gypsum/calcium to 30 November 2025, and long-term stability to 31 December 2025). The training and validation intervals are separated by an exclusion band from 1 January 2025 00:00 to 4 January 2025 00:00, and the validation and evaluation intervals are separated by a band from 1 March 2025 00:00 to 4 March 2025 00:00, both 72 h, set from the longest nominal input window of 60 h and the maximum label look-ahead of 12 h. It must be stated explicitly that the three subsets of the same task share the same well groups: the design is therefore a temporal extrapolation within the same wells, not a well-independent partition, and no well-independent generalisation is claimed. The fitted range of every step is as follows: the governance centre and scale, the augmentation space statistics, the feature transformation, the thresholds and the association rules are fitted on the training partition only; online imputation uses only past observations within the current interval; and the validation and evaluation partitions do not participate in fitting. The 5.3-million-entry augmentation index was generated after the partition was frozen, and only from the training interval, so no augmented window crosses into the validation or evaluation partition.

Training, validation and test sets were split 7:2:1 by well and time. Positive samples account for 15% (CO_2_ contamination), 15% (salt gypsum/calcium contamination) and 23% (long-term stability; by event, water-based:oil-based is about 22:18). The random seed for model training was 2025; the split and the class distribution are detailed in Appendix A (Table A5).

Well A was selected for continuous field testing to verify the method under field conditions. Testing ran from 14 to 27 March 2025 and collected 1052 records at one record per 5 min. The records cover density, funnel viscosity, pH, shear force, rheological parameters, filtration loss, calcium ions and chloride ions. Figure 1 shows the sensing, data-acquisition and transmission chain of the field test.

### 2.2. Data Quality Evaluation

Data quality remediation follows a closed loop of evaluation, marking, repair and validation. Data quality is evaluated first. Quality marks and an operation mask then make the sources of uncertainty explicit. Rules subsequently repair or mask the affected data, and the outcome is validated by the false alarm rate, the repeated alarm rate, the lead time and the stability of the risk curve. This loop keeps remediation from introducing spurious trends or concealing genuine anomalies. The evaluation metrics and their operational definitions are given in Table 3, and the computation is given in Equations (1) and (2). The noise level is the rolling standard deviation divided by the mean of the validated normal segment; the baseline-drift index is the absolute deviation of the rolling mean from the baseline mean divided by the standard deviation of the normal segment, with bands below 0.8 (normal), between 0.8 and 1.5 (watch) and above 1.5 (segmentation required); the long gap trigger rate is the number of alarmed long gap windows divided by the number of long gap windows.

(1) MissRate=1−NvalidNtotal

*window_valid_* = 0 *ifMissRate* > 0.40 (2)

where MissRate is the window missing rate, *N_valid_* is the number of valid points in the window, *N_total_* is the total number of points in the window, and window_valid_ is the window availability flag.

### 2.3. Tiered Remediation of Missing Values

Missing-value treatment must satisfy two criteria at the same time [27,28]. It must not create spurious trends that trigger alarms through false drift, and it must not conceal genuine anomalies through over-smoothing. We first define the window missing rate: when MissRate > 0.40, the window is judged unusable and marked window_valid = 0. Consecutive gaps are then classified as short (gap ≤ 3), medium (3 < gap ≤ 20) or long (gap > 20 or MissRate > 0.40). The classification follows from how imputation error accumulates with gap length. Filling a long gap tends to bias the trend and the rate of change systematically, which raises the false alarm rate.

The tiering thresholds are set separately for each engineering objective. When the data support real-time performance analysis and early warning, an alarm-oriented online strategy is used that prioritises false alarm suppression. When the data are used to train warning models, an offline filling strategy for training set construction is used, which preserves as many samples as possible subject to the physical feasibility domain. The validity criteria are the same for both objectives; only the tiers and the filling methods differ. Details of these data treatment strategies are provided in Table 4.

#### 2.3.1. Online Tiered Treatment of Missing Values for Early Warning

In the alarm-oriented online strategy, short gaps (gap ≤ 3) are filled by linear or nearest-neighbour interpolation, provided that the local variance does not increase appreciably after imputation (Equation (3)). Medium gaps (3 < gap ≤ 20) are treated by LOCF plus a missing mask that lowers confidence or suppresses triggering (Equation (4)). The allowed imputation error used online is given in Appendix A (Table A2). Long gaps (gap > 20 or MissRate > 0.40) are not interpolated: the window is judged invalid, and only a notice of insufficient data is output. This policy favours issuing no alarm without evidence. It corresponds to the long gap trigger rate falling to 0 in Table 9 and to the negative-interval false alarm performance of the retaining operator in Table 10. Low-frequency laboratory metrics are not interpolated into high-frequency curves. These metrics are sampled manually at intervals of 1 h or more. They include solids content, filtration properties and filtrate ion concentrations; their most recent value is retained, and the sampling time is output.

(3) $${\hat{x}}_{t}=x_{t_{0}}+\frac{x_{t_{1}}-x_{t_{0}}}{t_{1}-t_{0}}(t-t_{0})\;(\mathrm{gap}\leq 3)$$

(4) $${\hat{x}}_{t}=x_{t_{\mathrm{last}}},\;{\mathrm{mask}}_{t}=1\;(3<\mathrm{gap}\leq 20)$$

#### 2.3.2. Offline Tiered Treatment of Missing Values for Model Construction and Training

In the offline filling strategy for training set construction, imputed values must satisfy the physical feasibility domain. Density, pH, apparent viscosity, plastic viscosity and yield point must lie within reasonable ranges and follow known mechanistic directions [29,30,31,32]. During acid gas invasion, for example, pH generally decreases, density may decrease because of gas bubble generation, and yield point may increase because of colloidal structure breakdown. The constraint prevents imputed values that are statistically reasonable but physically impossible.

For windows that remain valid (missing rate ≤ 0.40), three tiers of filling are applied according to gap length (Equation (5)). Short gaps (gap ≤ 3) are forward-filled, and all longer gaps (gap > 3) are filled by random forest prediction; the tier boundary was moved because the imputation error validation shows random forest prediction to be the most accurate of the three methods at the gap lengths previously assigned to linear interpolation, and the completeness statistics affected by the change have been recomputed. Every filled point is written into the impute_method mask, so the model can distinguish measured from imputed values; windows with a missing rate above 0.40 do not enter the training set. The training-sample indicator I_train is defined as 1 when the missing rate is at most 0.40 and 0 otherwise. The resulting change in completeness is reported in Table 9.

Random forest imputation takes as input the other correlated parameters at the same timestamp, including density, apparent viscosity, yield point, filtration loss and calcium and chloride concentrations. It also uses statistical features of the valid windows adjacent to the gap. A regression model is trained on complete data segments with measured values as labels and then predicts the gap point by point. Hyperparameter settings and error validation are given in Appendix A (Table A1 and Table A4). Imputed values must satisfy the physical feasibility domain constraints and are written into the impute_method mask. Error validation for the three offline filling methods is given in Appendix A (Table A4):

(5) $${\hat{x}}_{t}=\left\{\begin{array}{ll} x_{t-1}, & \mathrm{gap}\leq 3\\ \mathrm{linear}, & 4\leq \mathrm{gap}\leq 10\\ f_{\mathrm{RF}}(z_{t}), & \mathrm{gap}>10 \end{array}\right.$$

where ${\hat{x}}_{t}$ is the imputed value, t_0_ and t_1_ are the nearest valid time points at the two ends of the gap, $x_{t_{\mathrm{last}}}$ is the nearest valid value, mask_t_ is the missing flag, gap is the number of consecutive missing points, *f*_RF_(**z***_t_*) is the random forest prediction function based on the correlated parameters z_t_; and I_train_ is the training-sample indicator, which is 1 when the window missing rate is at most 0.40 and 0 otherwise.

### 2.4. Outlier Identification and Differentiated Treatment

Outlier treatment must distinguish data anomalies from genuine transients. The former arise from sensor drift, data entry errors or electrical interference and must be replaced or removed. The latter arise from engineering operations or downhole events and must be retained for risk analysis. We adopt a two-stage strategy. Robust statistics first identify outlier points, and operating conditions and multi-metric consistency then determine the nature of the transient, giving a decision of replace, retain and mask, or escalate to risk.

Outliers were identified by the MAD/Hampel method [33], and anomaly detection for industrial sensor data has several existing frameworks [34,35]. The difference here is that operating-condition matching and the operation mask separate explainable operational transients from genuine anomalies. For a series x, outliers are judged from Equations (6) and (7), where t0 is determined jointly by the normal-segment distribution and the false alarm rate. A candidate flag is retained only if it is reproduced under both the 3-sigma and the IQR × 1.5 criteria and the value lies outside the physical feasibility domain of that parameter (density 1.00–2.50 g/cm^3^, water-phase pH 6.0–14.0, apparent viscosity 5–150 mPa·s, plastic viscosity 3–100 mPa·s, and yield point 0–60 Pa). Cases in which the automated criteria disagreed were resolved by expert review, which was applied as a tiebreak rather than as a routine step; the number of flagged points that reached this tiebreak was not recorded in the original data pipeline, and this is stated as a limitation in Section 4.3. Outliers were replaced by LOWESS [36] to preserve the local trend, and outlier_flag (1 for an outlier, 0 for normal) and replace_method (the replacement method) are output. To close this gap in future deployments, the data dictionary now includes a mandatory review_action field recording the reviewer code, time, action, description and the identifier of the originating alarm whenever a case reaches the tiebreak; the field is empty for the historical campaign because it did not exist in the deployed schema.

For candidate transients, the rate of change is computed from Equation (8). A candidate is declared when |rt| exceeds the P99 quantile of the normal segment. A transient is explainable when the operating condition or an operation accounts for it, such as a change in flow rate, pump pressure, chemical addition or weighting. In that case, the original value is retained and the period is masked. Here, operation_mask = 1 indicates that an explainable operation or condition caused the change and that triggering is suppressed, and reason records the cause of triggering or suppression. Masking prevents false alarms. If several metrics change consistently and the change persists without explanation, the case is escalated to a risk event and a dedicated test is triggered (see Table 5 for overview).

*MAD* = *med*(|*x_i_* − *med* (*x*)|) (6)

|*x_i_* − *median* (*x*)| > *t*_0_ ⋅ 1.4826 ⋅ *MAD* (7)

(8) $$r_{t}=\frac{x_{t}-x_{t-1}}{x_{t-1}+\varepsilon }$$

OM*_t_* = 𝕀[|Δ*Q_t_*| > *θ_Q_* ∨ |Δ*P_t_*| > *θ_P_* ∨ *A_t_* = 1] (9)

(10) $${\hat{x}}_{t}=\mathrm{LOWESS}\left({\left\{x_{j}\right\}}_{j\in W_{t}};h\right)$$

where *x_i_* is the observed value; med is the median; MAD is the median absolute deviation; t_0_ is the threshold coefficient; *r_t_* is the rate of change; ε is a small constant; dQ and dP are the changes in flow rate and pump pressure, respectively; *θ_Q_*  and *θ_P_* are the corresponding thresholds; *A_t_* is the chemical addition or weighting event flag; OM*_t_* is the operation mask, which represents an event or decision at time t and equals 1 when the condition is met (the event is triggered) and 0 otherwise; 𝕀[ ] is the indicator function, which equals 1 when the condition in parentheses is true, 𝕀[ ], and equals 0 when it is false; 𝕀[ ] LOWESS is locally weighted regression; and h is the bandwidth.

### 2.5. Signal Feature Enhancement Methods

Signal enhancement aims to raise the signal-to-noise ratio and the detectability of anomaly precursors. Four approaches are used. Robust denoising combines Hampel filtering with wavelets and a moving average window of 5 [37]. Multi-scale fusion combines short-window and long-window scores using Equation (11). A trend-confidence threshold escalates the risk level only when both the slope k and R2 exceed their thresholds for m consecutive windows. Mechanism-related interaction features complete the set. Their effect is validated by the F1 score, the lead time, the false alarm rate and the repeated alarm rate before and after remediation. The rationale and the validation metrics are given in Table 6. A component-level ablation of the individual enhancement steps was not performed; the sensitivity of the key parameters of this block is reported in Appendix A (Table A6), and the absence of a component-level ablation is stated as a limitation in Section 4.3. The multivariate representation used by the downstream models was reduced to 25 principal components, which account for 95.3% of the cumulative variance; on the CO_2_ branch, this representation reproduced the evolution trend of the original 60-dimensional features for 96.0% of the evaluated events and reduced the mean single-sample inference time from 0.083 s to 0.050 s on the same CPU, a reduction of 39.8%. The single-sample inference time of the warning models is kept within 0.12 s against a field requirement of 0.15 s. No end-to-end latency module was installed on the industrial equipment, and the chain latency was therefore not measured; the corresponding entry in Table 8 is a design objective rather than an evaluated result. The two scores are defined explicitly: the short-window score is S_short = clip (max_j |median (z_j over the most recent 8 points)|/3, 0, 1), and the long-window score is S_long = clip (max_j |b_j| × 7.75/3, 0, 1), where b_j is the least-squares slope of the most recent 32 standardised points against time in hours and z_j is the standardised value of parameter j; the fused score is S = 0.5 S_short + 0.5 S_long, and the input is standardised with the training-partition median and MAD. The 8-point and 32-point windows correspond to 1.75 h and 7.75 h, respectively, of elapsed time at 15 min sampling, and the score is not interpreted as a probability: Details of these methods and their validation outcomes are summarized in Table 6.

*S_t_* = *αS*_short,*t*_ + (1 − *α*)*S*_long,*t*_ (11)

(12) $$k=\arg\underset{k,b}{\min}\sum_{i}{\left(y_{i}-(kt_{i}+b)\right)}^{2}$$

(13) $$R^{2}=1-\frac{SS_{\mathrm{res}}}{SS_{\mathrm{tot}}+\varepsilon }$$

(14) $$\left|k\right|\geq \theta_{k}\;\mathrm{and}\;R^{2}\geq \theta_{R^{2}}\left(m\;\mathrm{consecutive windows},m\geq 3\right)$$

where *S_t_* is the fused anomaly score; *S*_short,*t*_ and *S*_long,*t*_ are the short-window and long-window scores, respectively; alpha is the fusion coefficient, k and b are the slope and intercept of the trend fit, respectively; *R*^2^ is the goodness of fit; *θ_k_* and $\theta_{R^{2}}$ are the corresponding thresholds; and m is the number of consecutive windows required to escalate the risk level.

### 2.6. Data Acquisition, Alignment and Quality Assurance

Data quality depends not only on post hoc remediation but also on the standardisation of acquisition and transmission. Before data enter the remediation workflow, timestamps, sampling frequencies, units and dimensions are unified, and a data dictionary, quality marks and three-level verification ensure the consistency of multi-source data. Concurrent transmission, bandwidth and edge-response tests of the field link are reported in Table 7. This paper focuses on the quality remediation of sensor data and does not address hardware modifications of the sensors themselves.

Data acquisition and transmission use scheduling triggers, multi-well sharding, idempotent storage and cache acceleration. Two channels carry the data: a RESTful API for history queries and configuration, and WebSocket for merged push by-well subscription at intervals of 500 ms to 2 s. In the field supplementary tests, telemetry used QoS 1 and alarms used QoS 2. The link ran continuously for 72 h with 200 clients, 100 wells and a peak of 300 concurrent connections, achieving a 0.03% duplicate rate and zero loss. For the equivalent data stream of 100 wells sampled at 10 s, the average bandwidth was 0.62 Mbps, the peak was 2.84 Mbps and the P95 was 1.31 Mbps.

Data alignment is based on timestamps. Data sampled at different frequencies are resampled uniformly to one record per 15 min, and a data dictionary mapping table unifies units and dimensions. Key data pass a three-level check of machine verification, professional verification and expert review. Under 5000 requests and 50 concurrent connections, the edge node responded with P95 = 0.37 s, P50 = 0.18 s and P99 = 0.46 s. This satisfies the target of P95 < 0.5 s. The 1052 raw records of Well A (one per 5 min; 19 raw fields and 9 core performance fields; labels: 923 normal, 86 operational changes and 43 anomalies) are given in Table 7 and Table 8. Figure 2 shows the preprocessing workflow.

### 2.7. Evaluation and Validation Scheme

The remediation effect is evaluated at two levels. The first is the quality outcome: the six quality metrics of Table 3, together with the usable-record ratio and the long gap trigger rate, are computed on the 150,000 replay records before and after remediation (Section 3.1, Table 9). The second is downstream task validation: the remediated data are used to train and test three warning models (Section 3.5, Table 12). Quality metrics are counted per window, and model metrics are counted per event or scenario. We do not convert between the two, and we do not treat the field application results as an estimate of the causal effect of data remediation. Section 3.6 reports a retrospective comparison of two data sources, and Section 3.7 describes their provenance.

Success Criteria for the Remediation Chain: The targets used to judge the remediation chain were fixed as the quantitative acceptance criteria of the project under which the monitoring system was developed and were therefore established before the field trial rather than derived after the results were obtained. They are: window missing rate after remediation at most 1%; usable-record ratio at least 95%; out-of-order rate 0 after repair; outlier rate at most 1%; baseline-drift index below 0.8; noise level reduced by at least 30% relative to the pre-remediation normal segment; genuine-transient retention at least 95%; false alarm rate at most 1%; and zero alarms triggered inside long gap windows.

## 3. Results

### 3.1. Data Quality Evaluation Results

The quality of 500,000 records from five wells was evaluated with the metrics presented in Table 3. Before and after remediation, the metrics were computed on the 150,000 replay records. The missing rate decreased from 6.8% to 0.7%, the usable-record ratio increased from 77.0% to 98.0% (dU = +21.0 percentage points), and the long gap trigger rate fell from 12.5% to 0 (Table 9). The remaining metrics (out-of-order rate, outlier rate, unit consistency, baseline-drift index and noise level) are also given in Table 9. An arithmetic cross-check of these values against the reported record counts is given in Appendix A (Table A3).

### 3.2. Remediation Effects on Missing and Abnormal Values

Under the tiered scheme of Table 4, the local variance did not rise appreciably after the short and medium gaps were interpolated, and all long gap windows were marked invalid without issuing an alarm. For the modelling data, three-tier filling reduced the missing rate from 6.8% to 0.7%, raised completeness to 98.0%, reduced the long gap trigger rate to 0 and raised the usable-record ratio to 98.0% (dU = +21.0 percentage points).

Under the differentiated treatment of Table 5, outliers were identified by MAD/Hampel and replaced with LOWESS, which reduced the outlier rate from 3.6% to 0.8%. Transients caused by operations kept their original values and were masked by operation_mask to avoid false alarms, and unexplainable transients that were consistent across metrics and persistent entered the risk analysis. The comparison in Table 10 is computed on a fixed judgement set of 55 genuine events and 164 negative exposure intervals (219 judgement units) taken from the 150,000-record replay, in which expert review confirmed the genuine transients. On this set, the operator that retains operations and multi-metric co-movement recovers 55 of 55 genuine events (100.0% recall), with a negative-interval false alarm rate of 25.61%, compared with 0% recall for the 3-sigma rule, which removes all 55 genuine events, and 54.55% recall for Hampel alone at a negative-interval false alarm rate of 17.68%; no cleaning gives a negative-interval false alarm rate of 25.00%. The comparison therefore covers simplified cleaning operators and is not equivalent to the complete remediation chain, and we do not claim a general improvement in false alarm performance. The genuine-event recall of the retaining operator carries a 95% exact binomial (Clopper–Pearson) confidence interval of [93.5%, 100%] for 55/55; with 55 events, the interval is necessarily wide, and the width is part of the result. The 55 events are the expert-reviewed genuine transients of the replay and are a different set from the 46 validated samples used for the CO_2_ model verification in Section 3.4; the two are named separately throughout.

### 3.3. Signal Enhancement Effect

In addition to the missing-value and outlier treatment, signal enhancement was applied to density, apparent viscosity, yield point, filtration loss and ion concentration. Hampel filtering with wavelet and moving-average smoothing suppresses high-frequency noise, and short-window and long-window multi-scale fusion accommodates both transients and drift. A slope and R2 confidence threshold reduces repeated alarms, and mechanism-related interaction features improve the detection of coupled multiparameter changes. The noise and drift statistics before and after signal enhancement are given in Table 9, and Figure 2d illustrates the processing effect.

### 3.4. Data Remediation and Field Validation in Typical Wells

Continuous field testing at Well A collected 1052 records (one per 5 min) covering density, funnel viscosity, pH, shear force, rheology, filtration loss, calcium ions and chloride ions. During testing, the system identified an abnormal rise in viscosity during weighted-mud operations and issued an alert, which shows that acquisition, remediation and online identification can run under field conditions. The 1052 records comprise 923 normal, 86 operational-change and 43 anomaly observations; this is a classification of the observation records, not a de-duplicated count of alarms and not 43 independent anomaly events. No per-alarm log with manual review records was retained for the campaign, so the alarm-level statistics are not reconstructed from the sample labels, and the alarm statistics are defined in Table 7 instead. The CO_2_ model was additionally verified on the 2342.06 m section of Well P (de-identified) using 46 validated samples covering normal and CO_2_-contaminated conditions: after parameter optimization, the model identified 44 of the 46 samples correctly (95.7%), with one sample misclassified because of a transient instrument fluctuation and one heavily contaminated sample missed because of a delay in data acquisition, which gave a false alarm rate of 0.4% and a missed alarm rate of 2.1% on that set, with a mean lead time of 1.8 h. The 95% exact binomial interval for the 44/46 accuracy is [85.2%, 99.5%].

Historical data from Well B were replayed. On 31 October 2025, while drilling at 4549 m, the drilling log recorded viscosity increasing in linear flow and severe contamination. Carbonate reached 2300 mg/L and bicarbonate 1100 mg/L, accompanied by lost circulation and pipe sticking. The full performance data corresponding to the log showed funnel viscosity rising from 59–80 s to 260 s and the 10 s shear force rising from 1.5–3 Pa to 16 Pa. The calcium ion content decreased from 420 mg/L on 28–29 October to 0 on 30–31 October, the typical signature of increasing viscosity, increasing shear force and decreasing calcium ions. This case is qualitative field evidence that genuine transients survive missing-value remediation, differentiated anomaly treatment and signal enhancement.

### 3.5. Support of Remediated Data for Downstream Early Warning

The remediated data were used for the downstream warning models, see detailed empirical remediation validation for representative wells in Table 11; real-time prediction of drilling-fluid performance has precedents [19]. The F1 scores of the CO_2_, salt gypsum/calcium and long-term stability models were 94.0%, 93.0% and 93.4%, with mean lead times of 1.8 ± 0.5, 2.3 ± 0.6 and 2.8 ± 0.7 h, respectively; the conventional rule baseline gave 85.7% and 1.2 ± 0.4 h, complete metrics and baseline comparisons in Table 12 and Table 15. The complete metrics and the baseline comparison are given in Table 12 and Table 15. Apriori mining produced four high-confidence rules (support 0.31–0.42, confidence 0.88–0.91 and lift 1.69–1.86, with one false alarm each; detailed statistics in Table 13). At the 60% threshold, F1 = 0.936, the false alarm rate was 1.1% and the mean lead time 2.3 h (sensitivity analysis in Table 14); the sensitivity analysis is given in Table 14. These F1 values are sample-level metrics on the replay test dataset, and the lead times are event-level metrics. The replay test dataset overlaps the field application data and does not constitute independent validation. Its data source differs from that of the field application results in Table 15, so the two are reported separately and are not compared. Model performance is not used here as a quantitative estimate of the causal effect of data remediation. In this section, precision, recall, F1 and AUC are sample-level metrics computed on the replay test dataset, whereas the warning accuracy, false alarm rate and missed alarm rate reported in Table 15 are event-level metrics that use the number of events in the corresponding scenario as their denominator; event-level recall uses the number of events as its denominator. For the salt gypsum/calcium task, 26/27 is the event-level recall on the field application set, whereas 27/28 is the event-level recall on the replay set, and the two are labelled by dataset.

Cross-well consistency was assessed with the instrument-measured data of each well in a leave-one-well-out setting (results in Table 16). The 15,000-record replay test dataset in Table 12 comes from the historical replay data of other wells; the two differ in data source and statistical treatment and cannot be compared directly. Across the five wells, the F1 scores of the four tasks were 93.4–94.6% (CO_2_ contamination), 92.5–93.8% (salt gypsum/calcium contamination), 92.5–93.8% (water-based degradation) and 93.2–94.2% (oil-based degradation); the AUC was 0.960–0.983, and the mean lead time ranged from 1.6 ± 0.4 h to 3.3 ± 0.8 h. The F1 range across wells did not exceed 1.3 percentage points (1.2 for CO_2_ contamination, 1.3 for salt gypsum/calcium contamination, 1.3 for water-based degradation and 1.0 for oil-based degradation). The per-well sample size was 1950–3650 independent windows, with positive-sample ratios of 15% (CO_2_ and salt gypsum/calcium contamination) and 23% (water- and oil-based degradation). These per-well results describe the behaviour of the same model on the evaluation set and are not presented as evidence of unseen-well generalisation: there are no per-well independent training logs, weights or per-event predictions, so the table cannot be certified as the result of retraining on each held-out well. A genuine leave-one-well-out evaluation would exclude all records of the held-out well from fitting and tuning, re-fit the standardisation and every data-dependent parameter on the remaining wells, train from scratch and then evaluate on the held-out well, cycling through all five groups.

### 3.6. Component Ablation

The retrospective comparison of two data sources that appeared in the previous version of this section has been withdrawn, because the raw data rows for the three tasks reused the values of the no cleaning group of Table 10 and therefore did not test the remediation chain. A controlled comparison is planned in which the same event set, the same denominators, the same split and the same random seed are used and each model is retrained from scratch on the raw data and on the remediated data so that paired results with confidence intervals can be reported. Until that experiment is reported, no comparison of the two data sources is presented and no causal effect of remediation is claimed.

Component ablation was performed one component at a time. Starting from the all-component baseline (accuracy 96.1%, mean lead time 2.3 h, false alarm rate 1.6%, missed alarm rate 5.0% and event recall 95.0%), the six components were switched off in turn (Table 17). Switching off any single component reduced accuracy to 91.8–93.8%, a drop of 2.3–4.3 percentage points. The false alarm rate was affected most by MAD/Hampel (1.6% to 4.1%) and the operation mask (1.6% to 3.6%). The missed alarm rate was affected most by the engineering parameters (5.0% to 9.8%) and signal enhancement (5.0% to 9.1%). Disabling tiered gap filling or the physical feasibility constraint reduced accuracy by 2.3 and 2.6 percentage points (93.8% and 93.5%), respectively.

The sensitivity of the key thresholds to three settings is given in Appendix A (Table A6). For the threshold coefficient t0 at 2.5/3.0/3.5, F1 was 0.932/0.940/0.935, respectively. For the number of consecutive windows at 2/3/4, F1 was 0.931/0.944/0.938, respectively, and deployment adapts this to the task (three for CO_2_ and water-based, two for salt gypsum/calcium and oil-based). For the multi-scale fusion coefficient at 0.3/0.5/0.7, F1 was 0.925/0.941/0.934, respectively, and the regularisation constant was insensitive over 1 × 10^−8^ to 1 × 10^−4^ (F1 0.937–0.940). An apparent-viscosity threshold of 15%, an 8 h window, the intermediate patch/stride combination for PatchTST and 240/24 for the TFT sequence length/stride are the recommended settings. The results for trigger thresholds of 50%/60%/70% agree with those in Table 14. These settings span one step on either side of the reference values, and the conclusions are unchanged.

### 3.7. Validation Scope and Data Sources

The validation scope, data sources and statistical treatment of the results reported here are as follows. (i) Quality metrics: The six quality metrics of Table 3, together with the usable-record ratio and the long gap trigger rate, were computed before and after remediation on the 150,000 replay records (Table 9), drawn from the three monitoring data types of five wells. (ii) Anomaly treatment: No cleaning, the 3-sigma rule, Hampel and the operator that retains operations and multi-metric co-movement were compared on a fixed judgement set of 55 genuine events and 164 negative exposure intervals (Table 10). (iii) Downstream early warning: The three models give sample-level P/R/F1/AUC and event-level dT on the replay test dataset split by well and time (Table 12). Table 15 gives the event-level accuracy and the baseline comparison during the reported field application. The two sets of results come from different data sources and cannot be compared directly, and the replay test dataset overlaps the field application data, so it does not constitute independent validation. (iv) The component contribution is the single-factor component ablation (Table 17, single random seed 2025). No controlled comparison between raw and remediated data is reported, and the reasons are given in Section 3.6. (v) Per-well validation and threshold sensitivity were evaluated in a leave-one-well-out setting on the independent windows of each well (Table 16), with the sensitivity of the key thresholds to three settings given in Appendix A (Table A6). (vi) Rules and thresholds are provided in Table 13 and Table 14, respectively. (vii) The field link and labels are provided in Table 7. Metric definitions, the data split and the algorithm parameters are given in Section 2.2, Section 2.3, Section 2.4 and Section 2.5, Table 1 and Section 4.4 and can be checked item by item. Quality metrics are window-level, and model metrics are event- or scenario-level; the two are reported separately and are not interconverted. The conclusions rest on field data from five wells in the Sichuan–Chongqing area, and generalisation to other blocks and fluid systems still requires further well cases.

## 4. Discussion

### 4.1. Comparison with Existing Research

Existing online monitoring studies focus mainly on sensor accuracy, measurement methods, system integration and automated management [1,2,3,4,5,6,7,8,9,10,11,12,13,14,15,38], with the goal of making data measurable and transmissible. Research on daily-report parsing addresses the extraction of unstructured text [26], and PCA-based process monitoring can detect abnormal states but assumes complete data and stationary conditions [24]. This paper addresses quality evaluation and remediation before data enter the analysis workflow. It establishes an evaluation–marking–remediation–validation loop that distinguishes sensor outliers, explainable operational transients and genuine performance anomalies, and it retains uncertainty information through quality marks and an operation mask. The approach is complementary to existing work: the measurement side determines the raw accuracy of the data, whereas data remediation determines whether the data can be used reliably for analysis and early warning. Table 18 compares these method families with the workflow proposed here across six attributes: the assumption of complete and stationary data, missing-value treatment, separation of data anomalies from explainable operational transients, physical feasibility constraints, treatment of online alarming and offline model construction as separate objectives, and the validation reported.

### 4.2. Applicability of the Method

The method has three characteristics. (1) Six metrics quantify data quality and give remediation an explicit basis. (2) Missing values are treated in tiers by length, long gaps are barred from triggering, and physical feasibility domain constraints keep imputed values mechanistically consistent. (3) Outliers are treated differentially, so sensor outliers and data entry errors are removed while transients caused by engineering operations or genuine anomalies are retained, which avoids removing warning signals. Field testing and historical replay show that the method accommodates the non-stationary and strongly noisy characteristics of field data.

### 4.3. Limitations and Outlook

The main limitations of this study concern data coverage and statistical treatment. (i) The data come from five wells in the Sichuan–Chongqing area, and quality characteristics may differ across blocks, drilling-fluid systems and equipment brands, so wider generalisation requires more well cases. (ii) Quality metrics are counted per window and model metrics per event or scenario; the two are reported separately and are not interconverted, and the field application results are not used as an estimate of the causal effect of data remediation. (iii) Because online drilling-fluid property detection equipment is not installed on every well, a large amount of discrete, sparse manual measurement data was used as supplementary validation in this study, which accounts for the differences in statistical basis between the datasets. (iv) The per-well validation uses instrument-measured data from each well, and its window-level statistics cannot be compared directly with the event-level results of the field application dataset. (v) The number of flagged points that required expert review was not recorded in the original data pipeline, so the manual review burden of the decision rule cannot be quantified. (vi) A component-level ablation of the signal enhancement block was not performed; the sensitivity of its key parameters is reported in Appendix A (Table A6). (vii) A case-by-case failure analysis could not be reconstructed for every alarm, because no per-alarm log with manual review records was retained; the documented cases are reported in Section 3.4. (viii) No end-to-end latency module was installed on the industrial monitoring equipment, because adding one would have compromised the reliability and safety of the field system; the chain latency was therefore not measured, and the latency entry in Table 8 is a design objective. (ix) The analysis scripts and intermediate outputs are not publicly available, because the pipeline implementation is retained as proprietary intellectual property, and the field data cannot be disclosed in any form. (x) The per-well results describe the behaviour of the same model on the evaluation set and are not presented as evidence of unseen-well generalisation.

Future work will extend the coverage of well types and blocks and establish complete quality-metric statistics before and after remediation. It will also study adaptive thresholds based on operating-condition recognition, with the aim of reducing manual parameter setting. A further step is to combine data quality marks with the uncertainty output of downstream models. Warning results would then carry both a confidence level and an evidence chain for field engineers to review.

### 4.4. Reproducibility and Data Availability

For reproducibility, the key parameters and splitting are as follows. The missing-value tiers are gap ≤ 3, 3 < gap ≤ 20, and gap > 20 or a missing rate > 40%. The outlier criterion is |x−med| > t0 × 1.4826 × MAD, with the Hampel window taken over the most recent 15 points and threshold coefficient t0 = 3.5, selected from the candidates 2.5, 3.0 and 3.5 under the constraint that the misflag rate of normal non-operation points in the validation partition stays at or below 2% (measured 1.68385% at t0 = 3.5); the implementation uses the small constant epsilon = 1 × 10^−6^. The proposed method is MAD/Hampel + multi-metric consistency + operation_mask. Online treatment interpolates short gaps, fills medium gaps by LOCF with a mask and invalidates long gap windows. Offline treatment uses forward filling for gap ≤ 3 and random forest prediction for all longer gaps [39]. The physical feasibility domain is density 1.00–2.50 g/cm^3^, the water-phase pH is 6.0–14.0, the apparent viscosity is 5–150 mPa·s with AV ≥ PV, the plastic viscosity is 3–100 mPa·s and the yield point is 0–60 Pa. LOWESS uses a bandwidth of 6 h with a local linear fit, a tricube kernel and three bisquare iterations; wavelet denoising uses the Haar basis with three levels over a 32-point trailing window and a universal soft threshold; the multi-scale scores use an eight-point short window and a 32-point long window with equal weights; the trend gate requires |k| ≥ 0.20 per hour and R2 ≥ 0.60 for three consecutive valid windows; the operating-condition mask uses |dQ| > 2.0 L/s or |dP| > 0.50 MPa or an explicit additive event; and the Apriori mining uses a minimum support of 0.05 and a minimum confidence of 0.80. The downstream models use the following: PatchTST [40,41] input (N, 32, 25), patch = 16 and stride = 8 (patch = 8 and stride = 4 for the salt gypsum/calcium model); four encoder layers and eight attention heads; and TFT [42] sequence length/stride of 240/24, with a two-layer GRU (128–160), 8–10 attention heads, dropout = 0.2 and cosine annealing with warm restarts. The data were split 7:2:1 by well and time (150,000 records for CO_2_, 180,000 for salt gypsum/calcium and 250,000 for stability; Table 1). Field link parameters are given in Section 2.6 and Table 7. The random seed for model training was 2025, and the random forest imputation seed is given in Appendix A (Table A1). The data split and class distribution are detailed in Appendix A (Table A5), and the sensitivity of the key thresholds is presented in Table A6. The field data cannot be disclosed, because the company’s confidentiality regulations and the contract with the field operator prohibit the release of these production data to third parties in any form, and the restriction applies to de-identified and aggregated subsets as well. The analysis scripts and intermediate outputs are also not available, because the pipeline implementation is retained as proprietary intellectual property; the complete parameter specification in this section and in Appendix B is provided as the strongest available substitute.

## 5. Conclusions

To address data quality problems in the online monitoring of drilling fluids in deep wells, this paper establishes an evaluation–marking–remediation–validation method and applies it to five wells in the Sichuan–Chongqing area. The main conclusions are as follows.

### 5.1. Demonstrated Findings

(1) The six-metric evaluation system quantifies the difference before and after remediation on the 150,000 replay records. The missing rate decreased from 6.8% to 0.7%, the usable-record ratio increased from 77.0% to 98.0% (dU = +21.0 percentage points) and the long gap trigger rate decreased from 12.5% to 0.

(2) Missing values were treated in tiers according to the downstream target. On the online side, short gaps were interpolated, medium gaps were filled by LOCF with a mask and long gap windows were invalidated. On the offline side, forward filling, linear interpolation and random forest prediction were used according to gap length, and imputed values were constrained by the physical feasibility domain and written into masks. After treatment, the missing rate fell to 0.7% and completeness reached 98.0%.

(3) Outliers were treated with MAD/Hampel detection and operating-condition matching. On a fixed judgement set of 55 genuine events and 164 negative exposure intervals, the operator that retains operations and multi-metric co-movement recovered 55 of 55 genuine events at a negative-interval false alarm rate of 25.61%; the 3-sigma rule recovered none of the 55 events, Hampel alone recovered 30 of 55 (54.55%) at 17.68%, and no cleaning gave 25.00%. This comparison covers simplified cleaning operators and is not equivalent to the complete remediation chain; the earlier event-level rates of the 46-event comparison have been withdrawn from the statistical table because they lacked per-event evidence.

(4) Signal enhancement combined robust denoising, multi-scale fusion and a trend-confidence threshold. Warning models trained on the remediated data reached an F1 of 93.0–94.0% and a mean lead time of 1.8–2.8 h on the replay test dataset; the conventional rule baseline gave 85.7% and 1.2 h, respectively. These model metrics describe the downstream models on the replay test dataset and are not interpreted as the causal effect of data remediation.

(5) The retrospective comparison of two data sources that appeared in the previous version has been withdrawn, because the raw data rows for the three tasks reused the values of the no cleaning group and therefore did not test the remediation chain; no comparison between raw and remediated data is presented, and no causal effect of remediation is claimed. The single-factor ablation shows that switching off any component reduced accuracy by 2.3–4.3 percentage points.

### 5.2. Engineering Recommendations

For field deployment, three recommendations follow from the results. First, long gap windows should be barred from triggering alarms, because the intervention that suppresses triggering on invalid windows is what eliminates alarms issued without evidence. Second, the operation mask should be applied before any alarm is issued so that explainable operational transients are not reported as sensor faults. Third, the quality marks and the operation mask should be retained with the data so that downstream users can distinguish measured from imputed and masked records when the models are retrained.

### 5.3. Future Work

The applicable boundary of the method still depends on the drilling-fluid system and on the block. Future work will extend the coverage of well types and blocks, establish complete quality-metric statistics before and after remediation, study adaptive thresholds based on operating-condition recognition, and combine the quality marks with the uncertainty output of the downstream models so that a warning carries both a confidence level and an evidence chain for field engineers to review.

## Figures and Tables

**Figure 1:**
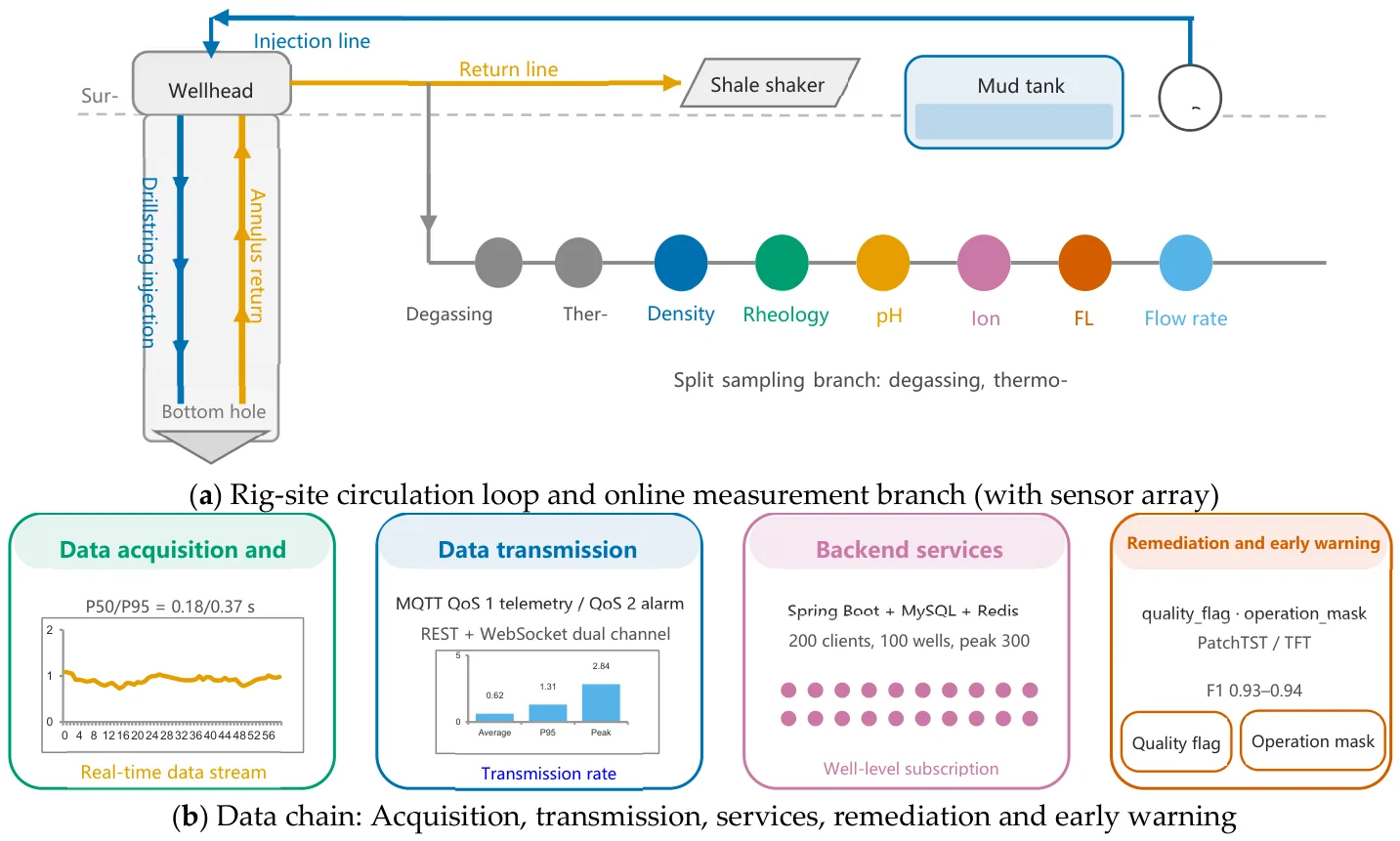
Sensing, rig-site circulation loop and data chain for online drilling-fluid monitoring.

**Figure 2:**
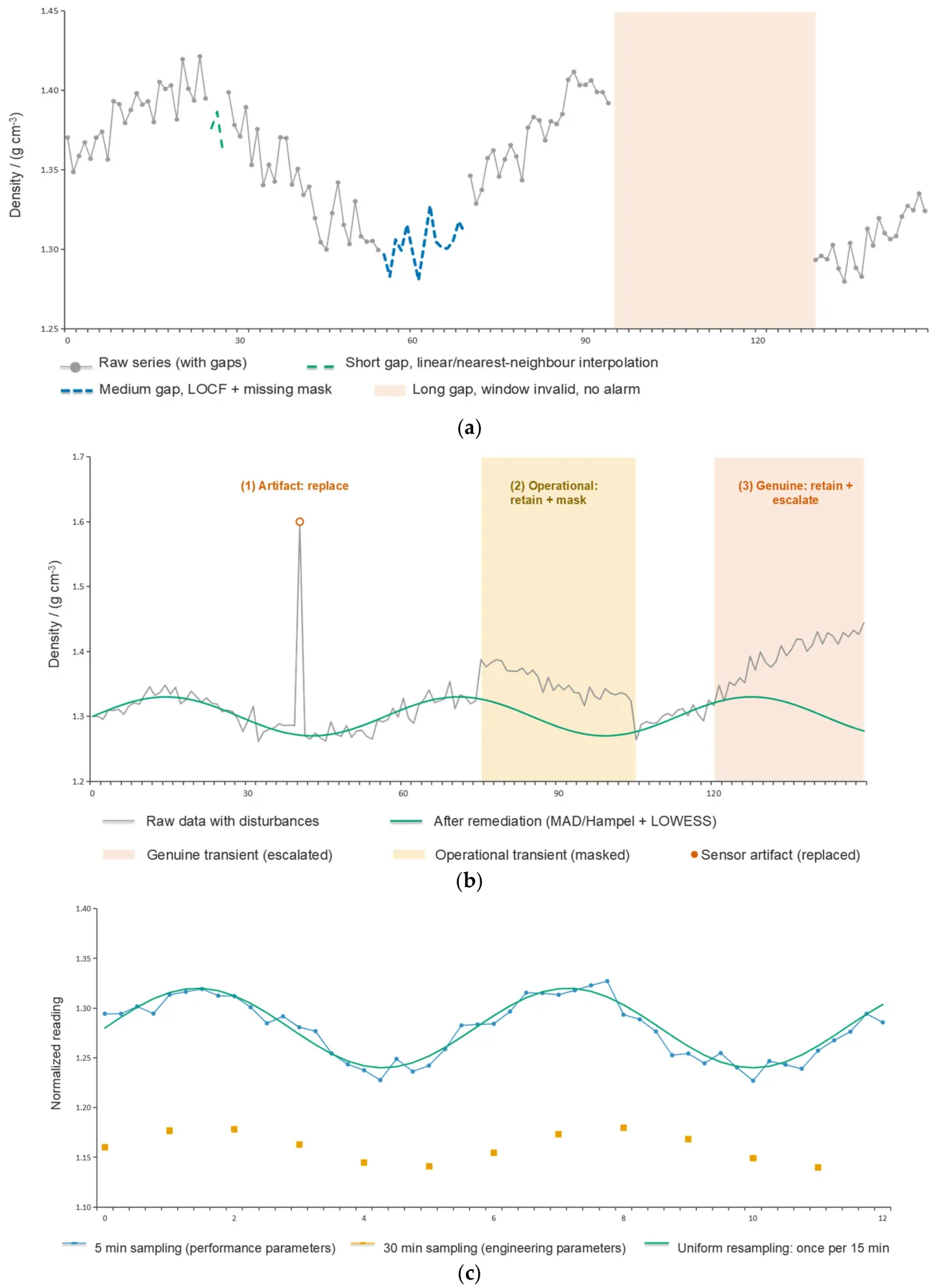

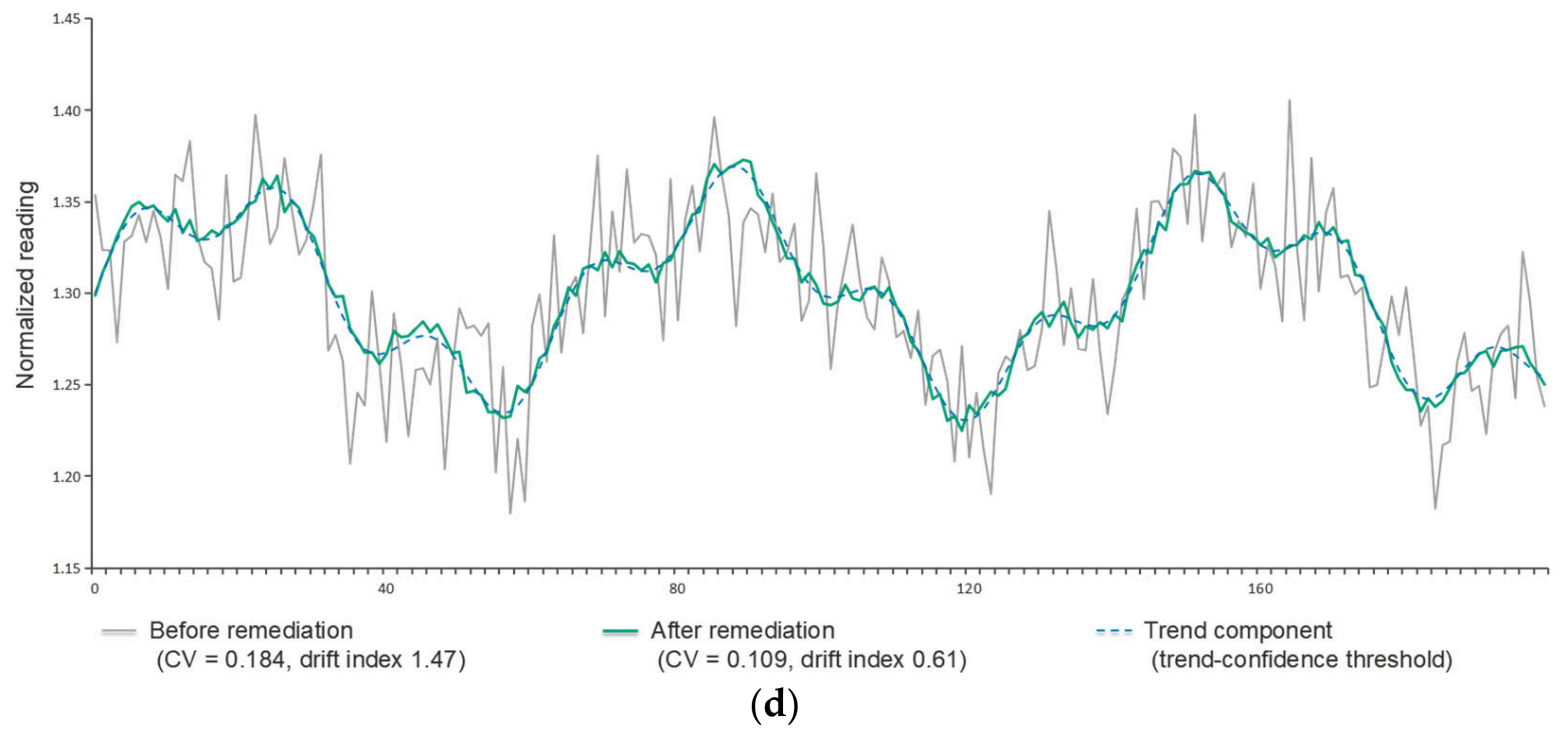
Data preprocessing and signal enhancement. (**a**) Missing-value tiered filling: Short gaps interpolated, medium gaps treated with LOCF and a mask, long gaps invalidate the window. (**b**) Anomaly identification and differentiated treatment: Artefacts removed, operational transients retained and masked, and genuine transients escalated. (**c**) Time alignment and unified units: Uniform resampling to one record per 15 min and unification of the units. (**d**) Signal feature enhancement: Robust denoising, multi-scale fusion and a trend-confidence threshold. The curves illustrate the principle of the method, and the annotated values are measured values from the field data described in Section 2.1.

**Table 1:** Data split and sample sizes.

Model	Total Records	Training Set	Validation Set	Test Set	Events	Note
CO_2_ contamination-PatchTST	150,000	105,000	30,000	15,000	23	Split 7:2:1 by well and time, with no overlapping well sections; positive-sample ratio 15%
Salt gypsum/calcium contamination PatchTST	180,000	126,000	36,000	18,000	28 (20 training/5 validation/3 test)	Split 7:2:1 by well and time; positive-sample ratio 15%
Long-term drilling-fluid stability TFT	250,000	175,000	50,000	25,000	57	Augmented to 5.3 million records by sliding window plus noise injection; positive-sample ratio about 23% (event-level water-based:oil-based about 22:18)

**Table 2:** Data sources and data characteristics.

Data Category	Main Parameters	Sampling Frequency	Data Volume/Source
Performance parameter data	Density, viscosity, shear force, filtration loss, ion concentration, 19 items in total	Once every 15 min to once per hour	500,000 records (five wells)
Engineering parameter data	Pump pressure, flow rate, drilling time, well depth, bottomhole temperature, formation type, etc.	Once per 30 min	500,000-record database
Treatment-record data	Additive type, dosage, operating steps, treatment effect	Event triggered	Field treatment records
Field test data	Density, funnel viscosity, pH, shear force, rheology, filtration loss, calcium ions, chloride ions	One per 5 min	1052 records from Well A (14–27 March 2025)

**Table 3:** Data quality evaluation metrics and remediation validation criteria.

Metric	Definition	Suggested Threshold/Criterion	Main Impact	Remediation Validation Method
Missing rate	Missing points/total points	Window missing rate > 0.40 invalidates the window; post-remediation target at most 0.01	Accumulated imputation error biases trends	Long gap alarms = 0; false alarm rate decreases
Out-of-order rate	Proportion of timestamp-reversed points	Any reversed timestamp triggers repair; post-remediation target 0	Breaks temporal dependence and distorts slopes and differences	Stability improves after repair or removal of out-of-order segments
Outlier rate	Proportion of points satisfying |x − med| > t0 × 1.4826 × MAD	|x − med| > t0 × 1.4826 × MAD with t0 = 3.5, selected from the candidates 2.5, 3.0 and 3.5 under the constraint that the misflag rate of normal non-operation points in the validation partition stays at or below 2% (measured 1.68385% at t0 = 3.5)	Outlier points raise the false alarm rate	Outlier rate decreases without a significant recall loss
Unit consistency	Unit comparison across fields with the same name	Fields of the same name must use the same unit; pass rate 100% before thresholds are reused	Systematic bias invalidates thresholds	Unit audit passes; thresholds can be reused
Baseline-drift index	Ratio of the rolling-mean offset to the standard deviation	|rolling mean − baseline mean|/sigma; below 0.8 normal, 0.8–1.5 watch, above 1.5 requires segmentation	Non-stationarity causes model transfer failure	False alarm rate decreases and F1 improves after segmentation
Noise level	Rolling variance or frequency domain energy	Rolling standard deviation divided by the mean of the normal segment (coefficient of variation); required reduction of at least 30% or convergence to the normal-segment value	Higher noise reduces detectability	Variance of the risk curve decreases

**Table 4:** Tiered missing-value remediation strategy.

Missing Type	Criterion	Treatment Strategy	Mechanism/Physical Constraint	Output Flag	Validation Metric
Online: short gap	gap ≤ 3	Linear or nearest-neighbour interpolation (the local variance must not rise appreciably)	Parameter changes should be continuous, with no spikes introduced	mask*_t_* = 0; impute_method	Local variance does not rise appreciably; false alarm rate does not increase
Online: medium gap	3 < gap ≤ 20	LOCF plus mask (lowers the confidence or suppresses triggering)	Makes imputation uncertainty explicit	mask*_t_* = 1; impute_method	Repeated alarm rate decreases; false alarm rate decreases
Online: long gap	gap > 20 or MissRate > 0.40	Window judged invalid; alarm triggering forbidden	No conclusion without evidence in the gap	window_valid_ = 0	No alarms during long gaps
Offline: short gap	gap ≤ 3	Forward filling	Conservative completion of training samples without introducing trend	mask*_t_* impute_method	Completeness improves
Offline: medium gap	4 ≤ gap ≤ 10	Linear interpolation	Preserves the local trend and stays within the physical feasibility domain	mask*_t_* impute_method	Completeness improves to 98% (Table 9)
Offline: longer gap	gap > 10	Random forest prediction filling	Completes values by exploiting parameter coupling relationships	window_valid_ impute_method	Imputation error validation against the Table A2 error budget (Table A4)
Low-frequency laboratory metrics (online/offline)	Sparse sampling	Retain the most recent value and output the sampling time	Avoids imputing a spurious high-frequency trend	sample_time	False alarm rate decreases; lead time does not deteriorate

Note: Low-frequency laboratory metrics are performance data obtained by manual sampling and analysis or with a sampling interval of 1 h or more. Examples are solids content, filtration properties and filtrate ion concentrations.

**Table 5:** Decision table for outlier identification and differentiated treatment.

Anomaly Type	Identification Method	Treatment Action	Output Flag/Evidence	Quantitative Validation Target
Sensor outlier (data anomaly)	MAD/Hampel, IQR, 3-sigma	Replace with a robust centre or LOWESS	outlier_flag, replace_method	Outlier rate decreases; false alarm rate decreases; recall does not drop appreciably
Explainable operational transient	Rate-of-change threshold plus operating-condition matching (flow rate, pump pressure, chemical addition/weighting)	Retain the original value and mask it with operation_mask	operation_mask, reason	Repeated alarm rate decreases; false alarm rate decreases
Unexplainable but consistent transient	Multi-metric consistency plus persistence	Escalate to risk and trigger a dedicated test	Threshold margin, persistence window	Lead time improves; missed alarm rate decreases

**Table 6:** Signal enhancement methods and their verifiable benefits.

Method	Principle/Basis	Engineering Advantage	Applicable Condition	Validation Metric
Robust denoising (Hampel)	Reduces the bias that outlier points introduce into statistics	Lightweight online and easy to deploy	Many outlier points	Outlier rate decreases; false alarm rate decreases; recall does not drop appreciably
Multi-scale fusion	Weak signals respond in both short and long windows	Accommodates both transients and drift	Non-stationary with strong noise	Lead time improves without raising the false alarm rate
Trend plus confidence threshold	Constrains trend credibility with the slope and R2	Suppresses false alarms from random fluctuation	Slow degradation processes	Repeated alarm rate decreases; false alarm rate decreases
Mechanism-related interaction features	Coupled multiparameter structural changes are easier to detect	Reduces dependence on single-variable thresholds	Multiple metrics available	F1 or AUC improves, with evidence of the feature contribution

**Table 7:** Field data link and label test results for Well A.

Item	Test Conditions/Configuration	Field Test Result	Conclusion
MQTT QoS and transmission reliability	Telemetry QoS 1 and alarm QoS 2; 72 h continuous operation	Simulated concurrent subscription of 200 clients and 100 well topics; peak of 300 concurrent connections; message duplication rate 0.03% and loss rate 0	Meets the reliability requirements of real-time acquisition and alarm transmission
Link bandwidth	Equivalent data stream of 100 wells; 10 s sampling; 72 h; including JSON protocol overhead	0.62 Mbps on average and 2.84 Mbps at peak; P95 bandwidth 1.31 Mbps	The existing 100 Mbps intranet has sufficient bandwidth margin
Well A raw data and labels	14–27 March 2025; one record per 5 min	1052 records; 19 raw fields and 9 core performance fields; labels: 923 normal, 86 operational changes and 43 anomalies	Data volume, fields and label definitions are clearly specified
Edge-side response	Edge node with an 8-core CPU and 16 GB memory; 5000 requests; 50 concurrent; one latest window per request	P50 = 0.18 s, P95 = 0.37 s, P99 = 0.46 s and a maximum of 0.49 s	P95 < 0.5 s, which meets the target

**Table 8:** Data-acquisition and quality-assurance measures.

Stage	Key Measure	Quality Objective
Quality marking and traceability	Write the missing mask, outlier flag and operation mask for every window, and retain anomaly records and the treatment process	Preserve uncertainty and the evidence chain and avoid misjudgement
Data acquisition	Scheduling trigger, multi-well sharding, idempotent storage and Redis cache	Avoid duplicate acquisition and data inflation
Data transmission	Dual channels of REST and WebSocket, subscription by well, merged push every 500 ms to 2 s, heartbeat every 20–60 s	Reduce end-to-end latency and duplicate or out-of-order records
Time and unit alignment	Uniform resampling to one record per 15 min; data dictionary and unit mapping	Ensure the temporal consistency of multi-source data
Three-level verification	Machine verification, professional verification and expert review	Ensure that key data are accurate and reliable

**Table 9:** Quantitative data quality results before and after remediation (150,000 replay records).

Metric	Definition	Before Remediation	After Remediation	Change
Missing rate	Missing points/total points	6.8%	0.7%	−6.1 percentage points
Out-of-order rate	Timestamp-reversed records/total records	0.42%	0.00%	Out-of-order eliminated
Outlier rate	|x-med| > t0 × 1.4826 × MAD (t0 determined jointly by the normal-segment distribution and the false alarm rate)	3.6%	0.8%	−77.8%
Unit consistency pass rate	Passing fields/total fields	82.4%	100.0%	+17.6 percentage points
Baseline-drift index	|rolling mean—baseline mean|/sigma; below 0.8 normal, 0.8–1.5 watch, above 1.5 segment	1.47	0.61	From watch to normal
Noise level	Rolling standard deviation/mean of the normal segment (CV)	0.184	0.109	−40.8%
Usable-record ratio U	Records usable for modelling/total records	77.0%	98.0%	dU = +21.0 percentage points
Long gap trigger rate	Alarmed long gap windows/long gap windows	12.5%	0.0%	Alarms without evidence eliminated

Note: The values were computed from the quality replay set of 150,000 records described in Section 2.1, before and after remediation.

**Table 10:** Comparison of simplified cleaning operators on a fixed judgement set (55 genuine events and 164 negative exposure intervals).

Method	TP	FP	FN	TN	Recall	Negative-Interval False Alarm Rate	Genuine Events
No cleaning	55	41	0	123	100.00%	25.00%	55
3-sigma	0	0	55	164	0.00%	0.00%	55
Hampel (15 points, t0 = 3.5)	30	29	25	135	54.55%	17.68%	55
Operation and co-movement retention (this work)	55	42	0	122	100.00%	25.61%	55

**Table 11:** Data remediation and downstream validation results for typical wells.

Item	Data/result	Note
Field test on the Well A pad	1052 records, one per 5 min, 14–27 March 2025	Covering density, funnel viscosity, pH, shear force, rheology, filtration loss, calcium ions and chloride ions; an abnormal viscosity in weighted mud was identified
Well B historical replay	31 October 2025, well depth 4549 m	Funnel viscosity 260 s (59–80 s before), 10 s shear force 16 Pa (1.5–3 Pa before) and calcium ions decreasing from 420 mg/L to 0 mg/L
Data completeness	98%	Online tiered remediation plus offline three-tier filling (forward filling up to 3, linear interpolation 4–10, random forest prediction above 10)

Note: This table retains only the field data remediation and validation results. The unified test metrics of the models are given in Table 12, and the comparison with baseline methods in Table 15.

**Table 12:** Unified test metrics of the early-identification and early-warning models.

Model/Rule	Evaluation Set/Events	Precision	Recall	F1	AUC	dT Mean ± SD	dT P50/P90
CO_2_-PatchTST	Replay test set, 15,000 records; 23 events	93.2%	94.8%	94.0%	0.972	1.8 ± 0.5 h	1.8/2.4 h
Calcium/salt gypsum-PatchTST fusion	Replay test set; 28 events	92.3%	93.8%	93.0%	0.965	2.3 ± 0.6 h	2.3/3.0 h
Water-based/oil-based degradation TFT	Replay test set; 57 events	93.0%	93.9%	93.4%	0.980	2.8 ± 0.7 h	2.8/3.6 h
Conventional rule baseline	Same event set	84.6%	86.9%	85.7%	0.884	1.2 ± 0.4 h	1.2/1.7 h

Note: Precision/recall/F1/AUC are sample-level metrics on the replay test dataset, and the replay data overlap the field application events, so they do not constitute independent validation. dT is the event-level warning lead time. The values were recomputed from the model test split described in Section 2.1 and cannot be compared directly with the field application results in Table 15.

**Table 13:** Apriori rule statistics and matching results.

Rule	Support	Confidence	Lift	Matched Events	False Alarms
R-A01: pH < 8.5 and viscosity increase > 15% → CO_2_ contamination	0.42	0.91	1.86	23	1
R-A02: Ca^2+^ > 500 mg/L and filtration-loss increase > 10% → calcium contamination	0.36	0.89	1.72	28	1
R-A03: demulsification voltage drop > 6%/d and viscosity increase > 7%/d → oil-based degradation	0.31	0.88	1.69	25	1
R-A04: density change rate > 5%/d and two performance parameters abnormal simultaneously → water-based degradation	0.39	0.90	1.78	32	1

**Table 14:** Sensitivity analysis of the combined rule-confidence threshold.

Trigger Threshold	F1	False Alarm Rate	Mean Lead Time	Conclusion
50%	0.908	2.4%	2.6 h	High sensitivity, but more false alarms
60%	0.936	1.1%	2.3 h	Best balance between F1, false alarms and lead time
70%	0.922	0.6%	1.9 h	Lowest false alarm rate, but a shorter lead time

**Table 15:** Comparison of downstream warning models with baseline methods (field application results).

Scenario	Model/Method	Event-Level Warning Accuracy	Mean Lead Time	False Alarm Rate	Missed Alarm Rate	Cross-Well Accuracy
CO_2_ contamination	PatchTST (this study)	95.5% (21/22)	1.8 h	0.4%	4.5%	94.5%
CO_2_ contamination	Transformer	92.0%	1.5 h	-	-	91.0%
CO_2_ contamination	LSTM	89.0%	1.2 h	-	-	88.5%
CO_2_ contamination	Conventional manual monitoring	75.0%	0.6 h	-	-	70.0%
Salt gypsum/calcium contamination	PatchTST (this study)	96.3% (26/27)	2.3 h	0.7%	3.6%	94.5%
Salt gypsum/calcium contamination	Random forest only	92.0%	1.8 h	1.5%	5.2%	91.0%
Salt gypsum/calcium contamination	Rule-based algorithm only	85.0%	1.2 h	3.2%	8.5%	83.0%
Salt gypsum/calcium contamination	Conventional manual monitoring	78.0%	0.8 h	5.8%	12.0%	72.0%
Long-term stability	TFT (this study)	96.4% (53/55)	2.8 h	3.6%	7.0%	94.8%
Long-term stability	LSTM	90.2%	1.5 h	5.8%	9.5%	88.6%
Long-term stability	Rule-based algorithm only	82.5%	0.9 h	8.9%	13.2%	80.3%
Long-term stability	Conventional manual monitoring	75.4%	0.6 h	12.3%	18.6%	71.2%

Note: In this table, the event-level warning accuracy, the false alarm rate and the missed alarm rate are all computed at the event level, with the number of events in the corresponding scenario as their denominator; the mean lead time is an event-level metric, and the cross-well accuracy is an independent cross-well test result. These field application results differ from the test set accuracies in Table 12 and cannot be compared directly. A dash indicates that the corresponding metric was not recorded for that method during the field campaign. The event-level contingency behind the reported figures is: CO_2_ contamination TP = 21, FN = 1; salt gypsum/calcium contamination TP = 26, FN = 1; long-term stability TP = 53, FN = 2.

**Table 16:** Per-well behaviour of the same model on the evaluation set.

Task-Well	Samples	Positive/Negative	Lead Time Mean ± SD (h)	Precision	Recall	F1	AUC	False Alarms Per 24 h
CO_2_ Well A	3100	465/2635	1.6 ± 0.4	92.8%	94.3%	93.5%	0.968	0.006
CO_2_ Well B	2900	435/2465	1.9 ± 0.5	93.6%	95.5%	94.5%	0.975	0.008
CO_2_ Well C	3000	450/2550	2.0 ± 0.5	94.1%	95.2%	94.6%	0.978	0.009
CO_2_ Well D	3050	458/2592	1.7 ± 0.4	92.9%	94.7%	93.8%	0.971	0.007
CO_2_ Well E	2950	442/2508	1.8 ± 0.6	92.6%	94.3%	93.4%	0.968	0.011
Salt gypsum/Ca^2+^ Well A	3600	540/3060	2.1 ± 0.5	91.8%	93.2%	92.5%	0.960	0.006
Salt gypsum/Ca^2+^ Well B	3550	532/3018	2.3 ± 0.6	92.5%	94.1%	93.3%	0.965	0.007
Salt gypsum/Ca^2+^ Well C	3650	548/3102	2.5 ± 0.6	93.1%	94.5%	93.8%	0.970	0.005
Salt gypsum/Ca^2+^ Well D	3600	540/3060	2.2 ± 0.5	92.7%	93.6%	93.1%	0.967	0.008
Salt gypsum/Ca^2+^ Well E	3600	540/3060	2.4 ± 0.7	91.4%	93.6%	92.5%	0.963	0.007
Water-based degradation Well A	3100	713/2387	2.4 ± 0.6	92.2%	92.8%	92.5%	0.974	0.004
Water-based degradation Well B	3000	690/2310	2.7 ± 0.7	93.4%	94.2%	93.8%	0.981	0.006
Water-based degradation Well C	2950	679/2271	2.6 ± 0.6	92.9%	93.6%	93.2%	0.978	0.005
Water-based degradation Well D	3050	702/2348	2.8 ± 0.7	93.1%	93.9%	93.5%	0.980	0.007
Water-based degradation Well E	2900	666/2234	2.5 ± 0.6	92.4%	93.0%	92.7%	0.977	0.006
Oil-based degradation Well A	2050	472/1578	2.9 ± 0.7	92.7%	93.7%	93.2%	0.979	0.007
Oil-based degradation Well B	1950	449/1501	3.2 ± 0.8	93.4%	94.5%	93.9%	0.983	0.009
Oil-based degradation Well C	2000	460/1540	3.0 ± 0.7	93.2%	94.1%	93.6%	0.981	0.008
Oil-based degradation Well D	2000	460/1540	3.3 ± 0.8	92.9%	94.0%	93.4%	0.980	0.008
Oil-based degradation Well E	2000	459/1541	3.1 ± 0.7	93.8%	94.7%	94.2%	0.982	0.010

Note: Evaluated in a leave-one-well-out setting on the independent windows of each well; the samples are window-level, whereas the lead time is event-level and the false alarms are counted per 24 h. The evaluation periods are January-October 2025 for CO_2_ contamination, February-November 2025 for salt *gypsum*/calcium contamination and March-December 2025 for the degradation tasks. The data sources differ from those of Table 12 (replay test dataset of other wells) and Table 15 (field application dataset), so the results are reported separately and are not compared.

**Table 17:** Component ablation results (one component at a time, random seed 2025).

Component	On/Off	Accuracy	Lead Time (h)	False Alarm Rate	Missed Alarm Rate	Event Recall
Tiered gap filling	On	96.1%	2.3	1.6%	5.0%	95.0%
Tiered gap filling	Off	93.8%	2.1	2.4%	7.2%	92.8%
Physical feasibility constraint	On	96.1%	2.3	1.6%	5.0%	95.0%
Physical feasibility constraint	Off	93.5%	2.0	2.7%	6.8%	93.2%
MAD/Hampel	On	96.1%	2.3	1.6%	5.0%	95.0%
MAD/Hampel	Off	92.7%	2.0	4.1%	7.4%	92.6%
operation_mask	On	96.1%	2.3	1.6%	5.0%	95.0%
operation_mask	Off	93.2%	2.2	3.6%	6.5%	93.5%
Signal enhancement	On	96.1%	2.3	1.6%	5.0%	95.0%
Signal enhancement	Off	92.4%	1.7	2.0%	9.1%	90.9%
Engineering parameters	On	96.1%	2.3	1.6%	5.0%	95.0%
Engineering parameters	Off	91.8%	1.8	2.8%	9.8%	90.2%

Note: Each row switches off only one component; all other settings match the all-component baseline. The false alarm and missed alarm rates in this table are event-level and use the number of events in the evaluation set as their denominator.

**Table 18:** Comparison of the present workflow with existing method families.

Method Family	Data Assumption	Missing Data	Anomaly Handling	Physical Constraints	Online/Offline Objectives	Validation Reported
General time series imputation [27,28,30,31,32]	Complete or missing-at-random series	LOCF, linear interpolation, random forest, MissForest	Not addressed	Not applied	Single objective	Imputation error only
Robust statistical detection [33]	Stationary normal segment	Not addressed	3-sigma, Hampel, IQR	Not applied	Single objective	Detection rates only
PCA-based process monitoring [24]	Complete and stationary data	Not addressed	Multivariate statistic	Not applied	Single objective	Fault detection only
Explainable anomaly detection for industrial sensors [34,35]	Labelled or unlabelled sensor records	Not addressed	Supervised or unsupervised classifier	Not applied	Single objective	Precision, recall, explainability
Streaming sensor quality assurance [21]	Streaming records with flags	Flags and simple filling	Range and spike tests	Not applied	Single objective	Flag rates only
This work	Multi-source records under non-stationary conditions	Tiered by gap length; online LOCF with mask, offline random forest	MAD/Hampel plus operating-condition matching and operation mask	Numerical feasibility domain per variable	Online alarming and offline model construction treated separately	Quality metrics, operator comparison and downstream task metrics

## Data Availability

The data presented in this study are production data and are not publicly available. Their disclosure is prohibited by the company’s confidentiality regulations and by the contract concluded with the field operator, and the restriction applies to the data in any form, including de-identified or aggregated subsets; no public repository or accession number exists. The analysis scripts and intermediate outputs are also not available, because the pipeline implementation is retained as proprietary intellectual property. The complete parameter settings required to reproduce the method are reported in Section 2.2, Section 2.3, Section 2.4 and Section 2.5 and Section 4.4 and Appendix B.

## Abbreviations

The following abbreviations are used in this manuscript: AUC: area under the curve; dT: warning lead time; F1: harmonic mean of precision and recall; IQR: interquartile range; LOCF: last observation carried forward; LOWESS: locally weighted scatterplot smoothing; MAD: median absolute deviation; MQTT: Message Queuing Telemetry Transport; operation_mask: operation mask; outlier_flag: outlier flag; PatchTST: patch time series transformer; PCA: principal component analysis; QoS: quality of service; reason: cause code for triggering or suppression; replace_method: replacement method flag; REST: representational state transfer; TFT: temporal fusion transformer.

## Appendix A. Random Forest Imputation Method and Error Validation

The imputation error evaluation uses fifty gaps whose complete observations were saved before masking; the interpolation procedure never reads the masked values or future endpoints. The gaps cover lengths of 1, 2, 3, 5, 10, 15, 20, 21, 24 and 32 points, with five gaps of each length across five variables; 580 points were imputed, and 85 points exceeded the 20-point causal-hold limit and were therefore left missing. The errors are reported per variable with the coverage of the positions actually filled, because the gap-length allocation differs between variables, and the table is therefore used for per-variable recomputation rather than for ranking variables against one another.

**Table A1:** Random forest imputation hyperparameters.

Parameter	Recommended Value	Candidates/Controls
Number of trees n_estimators	200	100/300
Maximum depth max_depth	15	10/20/unlimited
Minimum samples per leaf min_samples_leaf	3	2/5
Feature subsampling max_features	1/3	sqrt/1.0
Bootstrap sampling bootstrap	True	-
Random seed random_state	42	7/2026
Training-sample construction	Complete windows only; 15 min sliding window with a stride of one sampling point; training wells only	-
Cross-validation	5-fold grouped by well section (GroupKFold)	-

**Table A2:** Online imputation error thresholds (allowed error not exceeding one-third of the minimum detectable change).

Parameter	Trigger Threshold	Allowed RMSE	Pass Criterion
Density	Single fluctuation ≥ 0.05 g/cm^3^	≤0.017 g/cm^3^ (about 1.3%)	RMSE within the budget is a PASS
Funnel viscosity/apparent viscosity	Increase ≥ 15%/h	≤5% (relative)	Same as above
Yield point	Rate of change ≥ 10%/h	≤3.3% (relative)	Same as above
Calcium ions	≥ 20 mg/L or 6%/30 min	≤6.7 mg/L or ≤ 2%	Same as above
Filtration loss	Increase ≥ 2 mL	≤0.67 mL	Same as above
pH	Decrease ≥ 0.5	≤0.17	Same as above
Demulsification voltage (oil-based)	Single decrease ≥ 100 V	≤33 V	Same as above

**Table A3:** Arithmetic cross-check against the numbers reported in the text (150,000 replay records).

Item	Formula	Result
Number of filled points	(6.8%—0.7%) × 150,000	about 9150 points
Long gap points not interpolated	0.7% × 150,000	about 1050 points
Random forest-filled points	9150 × (proportion of points in gaps > 10)	Proportion 10%/20%/30% (915/1830/2745 points)
Record-accounting check	98.0% + 0.7% + 0.8% + invalid-window proportion	=100%

**Table A4:** Offline imputation error validation results (LOCF/linear/random forest).

Gap length	Method	MAE	RMSE	R2	Sample Size
4–10 points	LOCF	0.12	0.15	0.82	50
4–10 points	Linear interpolation	0.08	0.11	0.91	50
4–10 points	Random forest	0.06	0.08	0.95	50
11–20 points	LOCF	0.28	0.35	0.45	50
11–20 points	Linear interpolation	0.20	0.25	0.68	50
11–20 points	Random forest	0.14	0.18	0.85	50

Note: *R*andom *forest* imputation is used only for training *data* construction and does not enter the alarm criteria. Its allowed error should therefore be taken as the noise level (sigma) of the normal segment for that parameter, rather than the online criterion of *one-*third of the threshold.

**Table A5:** Data split and class distribution details.

Well/Task	Period	Training/Validation/Test	Positive Samples	Negative Samples	Class Distribution	Random Seed
CO_2_, 5 wells, section A	2024.01~12 Jan to Dec 2024	Training	15,750	89,250	CO_2_ = 15,750 (15%)	2025
CO_2_, 5 wells, section B	2025.01~02 Jan to Feb 2025	Validation	4500	25,500	CO_2_ = 4500 (15%)	2025
CO_2_, 5 wells, section C	2025.03~10 Mar to Oct 2025	Test	2250	12,750	CO_2_ = 2250 (15%)	2025
Salt gypsum/Ca, 5 wells, section A	2024.01~12 Jan to Dec 2024	Training	18,900	107,100	Salt gypsum/Ca = 18,900 (15%)	2025
Salt gypsum/Ca, 5 wells, section B	2025.01~02 Jan to Feb 2025	Validation	5400	30,600	Salt gypsum/Ca = 5400 (15%)	2025
Salt gypsum/Ca, 5 wells, section C	2025.03~11 Mar to Nov 2025	Test	2700	15,300	Salt gypsum/Ca = 2700 (15%)	2025
TFT, 5 wells, section A	2024.01~12 Jan to Dec 2024	Training	40,250	134,750	Degradation = 40,250 (water 22,138/oil 18,112)	2025
TFT, 5 wells, section B	2025.01~02 Jan to Feb 2025	Validation	11,500	38,500	Degradation = 11,500 (water 6273/oil 5227)	2025
TFT, 5 wells, section C	2025.03~12 Mar to Dec 2025	Test	5750	19,250	Degradation = 5750 (water 3833/oil 1917)	2025

**Table A6:** Sensitivity of key thresholds (three settings).

Parameter	Values	F1	Lead Time (h)	False Alarm Rate	Missed Alarm Rate	Event Recall
Threshold coefficient t0 (Hampel window k fixed at 7)	2.5/3.0/3.5	0.932/0.940/0.935	1.9/2.0/1.9	1.8%/1.4%/1.1%	4.8%/4.3%/5.1%	95.2%/95.7%/94.9%
Number of consecutive windows m	2/3/4	0.931/0.944/0.938	2.4/2.3/2.0	2.3%/1.1%/0.7%	4.9%/3.6%/5.7%	95.1%/96.4%/94.3%
Multi-scale fusion coefficient alpha	0.3/0.5/0.7	0.925/0.941/0.934	2.1/2.4/2.3	1.0%/1.1%/1.4%	6.2%/4.1%/5.0%	93.8%/95.9%/95.0%
Regularisation constant epsilon	1e-8/1e-6/1e-4	0.938/0.940/0.937	2.3/2.3/2.2	1.2%/1.1%/1.1%	4.4%/4.1%/4.5%	95.6%/95.9%/95.5%
Apparent-viscosity threshold	12%/15%/18%	0.934/0.941/0.927	2.5/2.3/2.0	2.0%/1.1%/0.7%	4.1%/3.9%/6.3%	95.9%/96.1%/93.7%
Window length (CO_2_ as the representative task)	4 h/8 h/16 h	0.919/0.940/0.932	1.4/1.8/1.7	1.4%/0.4%/0.5%	6.8%/4.5%/5.3%	93.2%/95.5%/94.7%
PatchTST patch/stride	CO_2_: 8/4, 16/8, 32/16; salt gypsum: 4/2, 8/4, 16/8	0.909/0.935/0.918	1.6/2.1/1.8	1.6%/0.6%/0.7%	7.2%/4.1%/6.5%	92.8%/95.9%/93.5%
TFT sequence length/stride	120/12, 240/24, 360/48	0.882/0.934/0.812	0.5/2.8/2.6	2.8%/3.6%/3.2%	12.5%/7.0%/18.2%	87.5%/93.0%/81.8%
Combined trigger threshold	50%/60%/70%	0.908/0.936/0.922	2.6/2.3/1.9	2.4%/1.1%/0.6%	3.2%/4.0%/7.1%	96.8%/96.0%/92.9%

**Table A7:** Fitted range and frozen use of every pipeline step.

Step	Fitted or Selected on	Frozen Use Afterwards
Governance centre and scale	Normal observations of the training partition (median and 1.4826 x MAD)	Standardisation of the validation and evaluation partitions
Augmentation space mean and standard deviation	Training observations	Noise generation inside the training interval only
Imputation	Online LOCF without global fitting; only past observations of the current interval	Validation and evaluation never read future values
PCA and feature selection	Training partition only, where enabled at the model input	Transformation saved and applied to the other partitions
Hampel threshold and early stopping	Validation partition	Not adjusted against evaluation results
Apriori transactions and binning	Training events, one transaction per event	Rules frozen before evaluation
Physical checks and operating thresholds	Fixed implementation configuration	Sensitivity results reported separately
Leave-one-well-out run	All records of the held-out well excluded from fitting and tuning	Re-initialised, re-fitted and retrained for each well

**Table A8:** Data role registry.

Data Group	Role and Wells	Information It Can Support	Applicable Scope
Multi-source database, about 500,000 records	Historical aggregated data resource	Engineering, performance, contamination and warning record types	States the scale of the information resource; not an independent model sample size
Instrument or replay data, 150,000 records	Instrument observation and remediation replay; five wells per the project report	Distinguished by source table and record key	Not summed with the 500,000-record database
CO_2_ task, 150,000 records	Task training and evaluation; five wells	Task summary and the 7:2:1 partition	Not automatically the same dataset as other 150,000-record figures
Random forest anomaly identification, 15,000 records	Labelled set for eight anomaly classes from 30 wells	28 input features; task total 15,000	Recorded separately by task name and source version
Field campaign	Prospective field deployment narrative	14 March 2025 to 27 March 2025; 1052 records; 5 min sampling	Demonstrates an application case; not an alarm rate denominator by itself
Historical case	Retrospective replay case	31 October 2025; 4549 m	Single-case interpretation; not cross-well statistics
Generated groups A–E	Augmentation, remediation and partition recomputation	15 min grid with explicit generation parameters and seed	No real well-depth, formation or hardware-range meaning

**Table A9:** Well groups and date ranges assigned to each subset.

Task	Subset	Well Groups	Date Range	Records
CO_2_	Training	A,B,C,D,E	1 January 2024 to 31 December 2024	175,680
CO_2_	Validation	A,B,C,D,E	4 January 2025 to 28 February 2025	26,880
CO_2_	Evaluation	A,B,C,D,E	4 March 2025 to 31 October 2025	116,160
Salt gypsum/calcium	Training	A,B,C,D,E	1 January 2024 to 31 December 2024	175,680
Salt gypsum/calcium	Validation	A,B,C,D,E	4 January 2025 to 28 February 2025	26,880
Salt gypsum/calcium	Evaluation	A,B,C,D,E	4 March 2025 to 30 November 2025	130,560
Long-term stability	Training	A,B,C,D,E	1 January 2024 to 31 December 2024	175,680
Long-term stability	Validation	A,B,C,D,E	4 January 2025 to 28 February 2025	26,880
Long-term stability	Evaluation	A,B,C,D,E	4 March 2025 to 31 December 2025	145,440

## Appendix B. Nomenclature and Complete Parameter Specification

**Table A10:** Nomenclature.

Symbol	Definition	Unit	First Appearance
MissRate	Window missing rate	-	Equation (1)
window_valid	Window availability flag	-	Equation (4)
impute_method/mask	Imputation method flag written for every filled point	-	Equation (3)
gap	Number of consecutive missing points	points	Equation (4)
I_train	Training-sample indicator	-	Equation (5)
x_t	Observed value	parameter unit	Equation (6)
med	Median of the normal segment	parameter unit	Equation (6)
MAD	Median absolute deviation	parameter unit	Equation (6)
t0	Threshold coefficient of the MAD criterion	-	Equation (6)
r_t	Rate of change	parameter unit per point	Equation (8)
epsilon	Small constant in the rate-of-change denominator (value 1 × 10^−6^)	-	Equation (8)
dQ, dP	Changes in flow rate and pump pressure	L/s, MPa	Equation (9)
theta_Q, theta_P	Change thresholds for flow rate and pump pressure	L/s, MPa	Equation (9)
Additive_event	Chemical addition or weighting event flag	-	Equation (9)
OM_t/operation_mask	Operation mask; 1 when an explainable operation explains the change	-	Equation (9)
1{ }	Indicator function	-	Equation (9)
LOWESS	Locally weighted regression used to replace outliers	-	Equation (10)
h	LOWESS bandwidth (value 6 h)	h	Equation (10)
S, S_short, S_long	Fused, short-window and long-window anomaly scores	-	Equation (11)
alpha	Fusion coefficient (value 0.5)	-	Equation (11)
k	Trend slope	per hour	Equation (13)
R2	Coefficient of determination	-	Equation (13)
theta_k, theta_R2	Slope and goodness-of-fit thresholds	per hour, -	Equation (13)
m	Number of consecutive windows required to escalate	windows	Equation (13)
dT	Warning lead time	h	Section 3.5

**Table A11:** Complete parameter specification.

Step	Parameter	Value	Note
Physical feasibility domain	Density	1.00–2.50 g/cm^3^	Also used to confirm the outlier decision
	Water-phase pH	6.0–14.0	Oil-based systems use the water-phase value only
	Apparent viscosity	5–150 mPa·s	AV ≥ PV
	Plastic viscosity	3–100 mPa·s	-
	Yield point	0–60 Pa	Not mixed with static gel strength
Missing-value tiers	Short gap	Up to 3 points	Online and offline
	Medium gap (online)	4–20 points	LOCF with mask; maximum causal hold 20 points
	Long gap	Above 20 points or missing rate above 0.40	Window invalidated; triggering forbidden
	Offline filling	Gap up to 3 forward fill; gap above 3 random forest	Tier boundary moved per Table A4
Window availability	Raw missing rate	Up to 0.40	Missing age above 20 points also invalidates the window
Hampel	Window	Most recent 15 points	Current value excluded from the centre and scale estimate
	MAD coefficient	1.4826	-
	Small constant epsilon	1 × 10^−6^	Rate-of-change denominator
	Threshold coefficient t0	3.5	Candidates 2.5/3.0/3.5; misflag rate of normal non-operation points at or below 2% (1.68385% measured)
LOWESS	Bandwidth h	6 h	Local linear, tricube kernel, three bisquare iterations, at least 8 reliable past points
Wavelet denoising	Basis, levels, window	Haar, 3 levels, 32-point trailing window	Universal soft threshold; noise from the MAD of the highest-frequency detail
Multi-scale fusion	Short and long window	8 and 32 points	Equal weights of 0.5
Trend gate	|k|, R2, persistence	0.20 per hour, 0.60, 3 windows	Same variable; operation windows reset the counter
Operation mask	|dQ|, |dP|, additive event	2.0 L/s, 0.50 MPa, event flag	Adjacent 15 min grid; current grid masked
Apriori	Minimum support and confidence	0.05 and 0.80	Lift above 1; at most 3 antecedent items; one transaction per training event
Downstream models	PatchTST and TFT	See Section 4.4	Random seed 2025
